# The Russian Epidemic

**Published:** 1865-07

**Authors:** 


THE RUSSIAN EPIDEMIC.—The epidemic at St. Petersburg does not seem to be diminishing. By the last accounts the number of persons suffering from it amounts to from 300 to 350 daily, and the number of deaths to about 90. On the 23d of May there were 4,430 patients in the hospital, 364 new ones were admitted, 253, were sent away cured, and 97 died.

NEW, YORK, June 28.—A letter has been received at the Custom House here, addressed to the State Department by our Consul at Port Mahon, announcing that the Russian plague is extending westward more rapidly than is generally supposed, and advising that all carges arriving from Russia or Turkish ports be rigidly scrutinized before landing. The disease is said to be the same as that which visited London a century since.

